# Farm scale on the transformation of agricultural mechanization services:Evidence from China

**DOI:** 10.1371/journal.pone.0346500

**Published:** 2026-04-08

**Authors:** Qiufen Zheng, Jiacheng Liu

**Affiliations:** 1 School of economics and management, Bengbu University, Bengbu, P. R. China; 2 School of Public Finance and Taxation, Nanjing University of Finance & Economics, Nanjing, P.R. China; Sichuan Agricultural University, CHINA

## Abstract

Understanding the dynamics of agricultural mechanization services is essential for fostering the sustainable development of grain production in countries where small-scale farming predominates. This study analyzes data from 584 villages and 586 service providers across Jiangsu, Sichuan, and Jilin provinces in China to investigate the factors driving changes in agricultural mechanization services and their potential impacts. The findings indicate that the scale of land parcels significantly and positively influences the evolution of agricultural mechanization in China in recent years. Specifically, an increase in plot size leads to substantial reductions in costs and improvements in the efficiency of local services, thereby creating a viable alternative to cross-regional services. Therefore, while fully leveraging the comparative advantages of cross-regional services, we should accelerate the establishment of local service systems in key areas that primarily rely on non-cross-regional services. By optimizing the organizational structure of agricultural machinery services and innovating large-scale land management models, we can comprehensively enhance agricultural production and operational efficiency.

## 1. Introduction

Farm mechanization is a critical pathway to reducing costs and increasing efficiency in grain production, serving as a key driver of sustainable agricultural practices. Two primary approaches to mechanization exist: purchasing agricultural machinery directly or outsourcing to agricultural mechanization services (AMS). In low-and middle-income countries, direct machinery purchases are often economically unviable due to small farm sizes, making AMS the predominant method [1–3]. Scholarly consensus indicates that AMS development effectively boosts agricultural productivity while alleviating scale diseconomies and low mechanization levels among smallholders [4–8]. Moreover, AMS reduces capital constraints and sunk costs for small farmers [6,9,10]. Within this context, labor-saving machinery access—facilitated by outsourcing services—supports the continued viability of smallholder farming [11–12]. According to data from the China Agricultural Machinery Industry Yearbook,by 2021, professional agricultural machinery cooperatives in China serviced 58,998.71 km², accounting for 54.61% of total grain mechanized harvesting area.

Amidst massive rural labor migration and population aging, AMS sustains both grain production stability and agricultural productivity [13–15]. First, declining net returns from grain cultivation—driven by off-farm labor shifts and rising production costs—are mitigated by AMS through labor substitution at relatively low prices, thereby underpinning China’s grain security [16–17]. Second, the quantifiable nature of grain production stages enables higher mechanization levels than other crops, incentivizing cost-sensitive farmers to prioritize grain cultivation [18], thus ensuring sustainable output growth [19]. Rather than representing a single transformational change, mechanization’s broad appeal to farm households results from an accumulation of incremental, overlapping, complementary advantages. These include labor savings, reduced drudgery, convenience, increased speed and timeliness of operations, improved ability to manage weather-related risks, and reduced loss of grain during harvesting [1].

Since 2003, China’s AMS market expanded rapidly, with Cross-Regional Services (CRS) being particularly notable [18,20]. CRS involves mobile operators providing harvesting services across administrative boundaries, leveraging geographical and seasonal variations to spread fixed costs. In 2013, CRS coverage for grain harvesting peaked at 26.01 million hectares (28% of national sown area), stimulating farmer enthusiasm and securing grain production growth [18]. However, recent trends reveal not only a contraction in cross-regional services (CRS) but also the emergence of local service monopolies, where local services are measured by the share of farmland area receiving mechanized services within the county, including both the rental of local agricultural machinery and farmers’ use of self-owned machinery [21]. This transformation’s impact on agricultural factor markets and production systems remains underexplored. By 2022, CRS harvesting area plummeted to 20,636.32 thousand hectares—a 43.8% decline. Analysis of 2017 farm household survey data from Henan Province further shows that 75.02%, 74.12%, and 73.79% of farmers used localized services for land preparation, sowing, and harvesting, respectively [22]. This raises a pivotal question: Why are agricultural machinery services becoming increasingly localized while CRS decline?

Existing literature attributes CRS dominance to comparative advantages in machinery investment, operational skills, and market scale, positioning it as a historical driver of agricultural growth [20]. Yet, scholars note CRS inefficiencies in meeting smallholders’ timeliness demands [23]. Conversely, localized service organizations leverage informational and social network advantages to better manage contingencies [24] and reduce transaction costs [25], catalyzing the CRS-to-local shift. Theoretically, this transition stems from dynamic changes in net advantages between service modes. As farm operations expand and plots become consolidated/regularized [26], localized services gain competitiveness by: (1) lowering coordination costs for contiguous plots, enhancing their transaction cost advantage; and (2) reducing inter-plot machinery movement costs, increasing effective operational capacity while mitigating scale disadvantages. Thus, localized services emerge as superior under land consolidation, explaining the macro-level shift from CRS contraction to local service expansion. Crucially, while CRS remains optimal for fragmented small-scale farming, scaled and contiguous plots make self-owned machinery or localized services economically preferable.

However, current research fails to integrate AMS market transformation with farmland market dynamics, resulting in two gaps: (1) inadequate explanation of endogenous drivers behind CRS decline and local service expansion, overlooking structural linkages between land and service scaling; and (2) insufficient analysis of the land-to-service transaction transition mechanism, obscuring trends toward horizontal integration in agricultural production.

This study’s contributions are twofold: First, we construct an analytical framework linking farmland scale to AMS market transformation, clarifying endogenous forces driving CRS decline and localized service growth to advance understanding of agricultural service evolution. Second, by framing the transition as market-level agent substitution—rather than merely price mechanisms—we provide new insights into the inevitability of AMS market restructuring.

## 2. Theoretical framework

### 2.1. Drivers of scale economies: A comparative study of CRS and Local Services

The differences in sources of economies of scale between CRS and local services are detailed in Table 1. CRS represent an innovative service model that has emerged from China's agricultural mechanization process. This approach leverages China's vast geographical span and the resulting temporal variations in crop planting, growth, and maturity across different regions. Service providers organize agricultural machinery to operate across county-level or higher administrative boundaries, enabling large-scale equipment owners to provide continuous services for up to seven months annually across more than ten provinces/municipalities. This mobile service model significantly reduces per-acre machinery costs [20].

**Table 1:** Differences in economies of scale sources between CRS and local services.

Index	Local services		CRS
	Self-purchasing machinery	Specialized Local Services	
market size	small	larger	largest
Total cost of market acquisition	low	higher	highest

CRS embodies the principles of specialized division of labor, with economies of scale serving as its fundamental theoretical basis. As market size expands, average total costs decrease. However, the market scale for CRS is constrained by two key factors: fixed agricultural land locations and the time-sensitive nature of farming seasons. These limitations affect the ability to spread fixed costs across sufficient acreage and annual working hours [23]. The model faces substantial transaction costs, including accommodation expenses for mobile operators, high negotiation costs among non-acquaintances, and operational risks stemming from inter-regional coordination failures and empty machine transfers. Economies of scale are only achievable when the rate of cost increase from market acquisition remains lower than the rate of market expansion.

Local services refer to farmers who own machinery providing services for themselves or for other farmers in the county. If farmers who own machinery mainly provide services for themselves, we call it as” self-purchasing machinery”. If farmers who own machinery mainly provide services to other farmers in the county, we call it as “Specialized Local Services”. Its main advantages are the vital timeliness of homework and low transaction costs [8,25]. Its disadvantage lies in the area's limited total scale of operations, which restricts reducing machine operation costs. Local service providers often transition to land-based operations for large-scale land management purposes. To ensure the workload matches operational capacity while internalizing transaction costs through vertical integration.

### 2.2. From CRS to Local: How Farmland Scaling Drives the Substitution of AMS

The advancement of agricultural mechanization in China, characterized by increasing farm operation scale and improved land plot regularity, has enhanced the suitability for mechanized production. These changes in production conditions have accentuated the comparative advantages of local service providers in terms of economies of scale. Land consolidation reduces coordination costs and improves machinery operational efficiency.

Under these conditions,CRS represent a rational choice for small-scale, fragmented landholdings. However, as farm sizes expand and land consolidation progresses, the economies of scale offered by local service providers or self-owned machinery become more pronounced. This leads to a gradual substitution away from CRS, as larger farms find it more feasible and cost-effective to use local machinery services or purchase equipment rather than rely on cross-regional service providers.

For illustrative purposes, we can consider the spectrum of mechanization options. Self-purchasing machinery typically involves the smallest operational scale and lowest transaction costs but requires bearing the full fixed cost of the equipment. Specialized local services offer an intermediate option. CRS can access the largest operational market but incur the highest transaction costs due to distance and coordination.

Therefore, the most direct mechanism by which land scaling reduces reliance on CRS is the increased adoption and economic viability of local machinery services and self-owned equipment. The consolidation and contiguity of plots further reinforce this effect by enhancing operational efficiency for local and self-owned machinery.

## 3. Materials and methods

### 3.1. Model

The paper aims to explore the impact of farmland large-scale management on the transformation of AMS. Firstly, verify the relationship between the farmland large-scale management and the reduction of CRS (or the increase of local services). The model is as follows.


$${\text{M}}_{\text{it}}=\alpha_{\text{0}}+\alpha_{\text{1}}{\text{P}}_{\text{it}}+\sum {\gamma_{\text{j}}\text{X}}_{\text{jit}}+\mu_{\text{i}}+\theta_{\text{t}}+\epsilon_{\text{it}}$$ (1)

Where $M_{it}$ is “the ratio of the service area to farmland area for CRS of village *i* in period *t* “. It is specifically measured by the proportion of *t*he operational area completed by CRS providers from other counties in the questionnaire, i.e., (operational area completed by CRS providers from other counties / total AMS area) × 100%.

$p_{it}$ is “the large-scale plot of the village *i* in period *t*. “Considering the availability of data and research needs, *t*hese indicators are selected to measure. For example, “the largest plot area of the village “, “the total area of plots with an area of over 15 mu(1 mu = 0.067 hectares)”and “the total area of plots with an area of over 30 mu”.

$X_{jit}$ is the control variable. Referring to Liu et al. [27], it includes economic level, demographic characteristics, nonfarm employment, etc (the explanations of these variables are detailed in Table 5). $\mu_{i}$ and $\theta_{t}$ denote individual and time effects, respectively. ${\;\varepsilon }_{it}$ is random disturbance terms.

Secondly, this paper explores the mechanism by which agricultural land scaling affects the reduction of CRS (or the increase of local services). According to the analysis framework, compared to CRS, the scaling of farmland is more conducive to achieving economies of scale in local services. Therefore, we examines from the perspectives of cost and efficiency. The model is as follows.


$${\text{N}}_{\text{it}}=\beta_{\text{0}}+\beta_{\text{1}}{\text{P}}_{\text{it}}+\beta_{\text{2}}{\text{CS}}_{\text{it}}+\beta_{\text{3}}{\text{P}}_{\text{it}}\cdot {\text{CS}}_{\text{it}}+\sum {\theta_{\text{j}}\text{Z}}_{\text{jit}}+\mu_{\text{i}}+\theta_{\text{t}}+\epsilon_{\text{it}}$$ (2)

$$E_{i}=\alpha_{\text{0}}+\beta_{\text{1}}P_{i}+\beta_{\text{2}}{CS}_{i}+\beta_{\text{3}}P_{i}\cdot {CS}_{i}+\sum {\gamma_{j}V}_{ji}+\mu_{i}$$ (3)

Where $N_{it}$ is “the cost per mu of service providers i in period *t* in equation (2).” The total service cos*t*/total service area of service organization i in period t. The two indicators of “total service cost of service organization i in period t” and “total service area of service organization i in period t” are part of the questionnaire.

$E_{i}$ is the efficiency of service providers *i* in period *t* in equation (3). This variable is measured using the BBC-DEA model.

$P_{it}\;$is” large-scale plot of service providers *i* in period *t*.” As it is measured from the perspective of service providers, *t*he area of mechanical patching operations by service providers is used to measure. For example, “total area of 30 mu contiguous operation plots,” “total area of 15 mu contiguous operation plots.”

${CS}_{it}$ is whether the service provider *i* provides CRS in period *t* (1 = yes; 0 = no). $P_{it}\cdot {CS}_{it}$ is the term for the in*t*eraction between $P_{it}$ and ${CS}_{it}$*. Z*_*jit*_. *V*_*ji*_ denotes other control variables that affect the cost per mu(or efficiency), including service providers’ characteristics, home characteristics, etc (the explanations of these variables are detailed in Table 6).

The estimation of equation (1) may be subject to endogeneity concerns. These primarily arise from two sources. On the one hand, the reduction of CRS may force land operators to expand their scale in order to digest excess agricultural machinery operating capacity. The scale of agricultural land management and the transformation of AMS may be mutually causal. On the other hand, despite controlling for a range of observable factors, some unobservable variables—such as farmer ability or entrepreneurial endowment—may simultaneously influence both the dependent variable and the core explanatory variable, leading to omitted variable bias. To address these concerns, this study employs instrumental variable (IV) estimation to mitigate potential endogeneity.

The instrumental variable selected for large-scale farmland operation is “the number of grain procurement points in the village five years prior.” The validity of this instrument is justified on two grounds. First, relevance: the historical presence of grain procurement points reflects the level of local supporting services for large-scale grain operations, which is correlated with the current scale of farmland management but unlikely to have a direct effect on the contemporary transformation of the AMS system. Second, exogeneity: as a historical variable measured five years before the survey period, it precedes both the outcome and explanatory variables in time, thereby reducing the risk of reverse causality and minimizing correlation with contemporaneous unobservable shocks.

### 3.2. Data source and sample description

The data used in this study are drawn from the “Follow-up Survey on Agricultural Production Outsourcing Service Organizations” conducted by the research group of the College of Economics and Management at Nanjing Agricultural University in 2014 and 2019. This paper primarily utilizes the village-level sample data and service organization sample data from this survey. The sampling design and implementation process are detailed below.

#### 3.2.1. Village-level sample sampling.

Based on China's three-tier administrative division system (Province-County-Township), a multi-stage sampling method was employed to successively select sample provinces, counties, and townships.

(1) **Sample Province Selection**

Comprehensively considering factors such as economic development level, grain production status, and regional representativeness, the provinces of Jilin, Jiangsu, and Sichuan were selected as the follow-up survey regions. The positioning is as follows:

Jilin: Represents major grain-producing regions characterized by abundant agricultural resources (especially arable land) and a high level of mechanization.

Jiangsu: Represents economically developed regions featuring dense populations, well-developed non-agricultural industries, and a mature agricultural outsourcing service market.

Sichuan: Represents economically underdeveloped regions characterized by scarce arable land resources, mountainous terrain, and a relatively underdeveloped agricultural outsourcing service market.

(2) **Sample County and Township Selection**

Within each sample province, three sample counties were selected using a stratified sampling method based on indicators like farmers’ net income per capita, resulting in a total of nine sample counties. Subsequently, two sample townships were selected from each sample county, totaling 18 townships.

(3) **Administrative Village Census**

A census was conducted covering all administrative villages within the 18 sample townships. The 2014 survey covered 331 villages, while the 2019 survey covered 319 villages (the 2014 and 2019 surveys actually collected data for the years 2013 and 2018, respectively). The number of villages successfully tracked in both survey waves was 297. After excluding 5 villages that had completely ceased grain cultivation by 2018, the final village-level sample for empirical analysis comprised 292 villages, yielding a total of 584 village-year observations across the two periods.

#### 3.2.2. Service organization sample.

The service organization sample was obtained through the following steps.

(1) Within each sample township, six administrative villages were selected using simple random sampling.
(2) Within the selected villages, 10–12 agricultural outsourcing service organizations were chosen from each village based on the local development of such organizations, following the principle of stratified random sampling. In total, 188 service organizations were surveyed across the 18 townships.

Special Notes

(1) The service organization questionnaire was administered only during the 2019 survey; it was not included in 2014.
(2) In the 2019 survey, retrospective data for key variables (e.g., service scope, service segments, and labor productivity) were collected for 2016 and 2017, aiming to construct a panel data structure.
(3) For information on grain‑harvesting services, the research group collected data for the years 2016–2018 (retrospectively for 2016 and 2017, current for 2018) from the 188 service organization samples during the 2019 survey, resulting in a total of 564 valid service organization-year observations over the three years.
(4) For the calculation of agricultural machinery service efficiency using the DEA method, data on total inputs and total outputs were required. Since retrospective data for 2016 and 2017 were not collected, the efficiency analysis section only utilizes the 188 service organization samples from 2018 (i.e., the current year data reported in the 2019 survey) for cross-sectional regression.
  3.2.3 **Data reliability and representativeness**

Although the data used in this study were not specifically designed for the current research topic, the data exhibit strong reliability and representativeness in capturing the development of the agricultural machinery service market, for the following reasons.

(1) **Data Reliability**

The survey was designed and implemented by a professional research team from Nanjing Agricultural University, with extensive experience in agricultural and rural surveys. Prior to the formal survey, pre‑tests and interviewer training were conducted to ensure a clear understanding of the questionnaire and consistency in data collection. Throughout the fieldwork, strict quality‑control measures were adopted, including on‑site verification and logic‑checking of questionnaires. For retrospective data (e.g., 2016 and 2017 information collected in 2019), enumerators used local events and agricultural calendars as memory aids to improve recall accuracy. Moreover, key variables (such as service area and machine ownership) were cross‑checked with village cadres and other service organizations where possible, enhancing the credibility of the data.

(2) **Regional Representativeness**

Jiangsu: As the region with the largest scale and longest history of inter-regional agricultural machinery operations in China (pioneering the “Southern Machines to Northern Deployment, Cross-Regional Operations” model), it represents developed areas with robust cross-regional operational capabilities. Its selection offers analytical foresight.

Jilin and Sichuan: Serving as comparative regions to Jiangsu, both are significant grain-producing areas, effectively reflecting machinery service development in such regions.

Jilin: Features large per capita arable land area and a remarkably high comprehensive mechanization rate for major crops (94%, approximately 20 percentage points above the national average). As a crucial national commercial grain supply base, Jilin contributes 6% of the nation's grain output and 10% of its commercial grain supply while accounting for only 4% of the national arable land, demonstrating significant representativeness.

Sichuan: Has experienced notably higher grain output growth rates than the national average in recent years. It exhibits far more acute man-land constraints and higher degrees of land fragmentation than Jiangsu and Jilin (with an average household arable land area of only 5.10 mu, significantly below the national average of 7.11 mu).

In summary, the sample regions cover a typical area for cross‑regional machinery operations (Jiangsu), a highly mechanized commercial grain base (Jilin), and a major grain‑producing region with acute man–land constraints (Sichuan), achieving diversified representativeness in terms of agricultural resource endowments and locational characteristics.

(3) **Survey Relevance and Depth**

This survey specifically targets agricultural machinery services. Its questionnaire delves deeply into information concerning the demand side (farmers/villages), the supply side (service organizations), and village‑level development of machinery services. Compared with publicly available, comprehensive large‑scale databases, this survey provides more granular and in‑depth information pertinent to agricultural machinery service issues.

Therefore, the dataset is not only well‑suited to the needs of this study but also constitutes a significant advantage in terms of data source reliability and representativeness.

### 3.3. Descriptive statistics

(1) **Macro Fact: Decline in Cross-Regional Services in China**

In recent years, with the rapid advancement of agricultural mechanization and the vigorous development of agricultural machinery services, China's comprehensive mechanization rate for plowing, planting, and harvesting of grain production has achieved remarkable growth. According to statistics, this indicator surged from 45.85% in 2008 to 73.11% in 2022, as documented in the China Agricultural Machinery Industry Yearbook.

During this process, the rapid expansion of cross-regional operations became particularly noteworthy. Taking cross-regional mechanical harvesting services for wheat as an example, the service area was only 5,372 thousand hectares in 2000 but soared to 14,426.7 thousand hectares by 2013, with an average annual growth rate of 7.9%. However, it is worth noting that after 2013, the total area of cross-regional machinery services continuously declined from 36,719 thousand hectares to 20,636.32 thousand hectares in 2022 (see Table 2), representing a cumulative drop of 43.8%. This trend indicates that China's cross-regional agricultural machinery service market has entered a contraction phase.

**Table 2:** Development Status of Cross-Regional Agricultural Machinery Services (2013–2022).

year	Total Area	Plowing Area	Sowing Area	Harvesting Area
2013	36719	6767	3085	26005
2015	25770.3	5264.75	2575.27	16522.9
2016	23386.9	4428.57	2159.91	15803.4
2018	20711.8	3898.62	1873.38	13747.5
2019	20478.7	3833.18	1946.79	13708.1
2020	19899.7	3751.84	1979.58	13356.7
2021	20603.04	3847.24	2067.32	13844.57
2022	20636.32	3810.79	2217.69	13843.18

Unit: thousand hectares

(2) **Micro-level evidence: Contraction of cross-regional services observed in parts of China, with marked localization trend in AMS**

Micro-level data also shows that while agricultural machinery services have developed rapidly and become widespread, CRS have declined in some areas. All three provinces saw significant growth in AMS levels from 2013 to 2018. Sichuan had the most dramatic increase, jumping from 29.22% to 73.86%, indicating rapid adoption of AMS. Unlike Sichuan, Jiangsu has shown a declining trend in CRS, dropping from 44.51% in 2013 to 43% in 2018. Additionally, Jilin's cross-regional services remained relatively stable, but the proportion was only 18.33%, indicating that localized agricultural machinery services are more common in the Jilin region (see Table 3).

**Table 3:** Development of AMS and Cross-Regional Services in Jiangsu, Sichuan, and Jilin.

Province	Type	2013	2018
Jilin	Ratio of AMS	59.00%	73.51%
	Ratio of CRS	18.33%	18.33%
Jiangsu	Ratio of AMS	63.25%	73.78%
	Ratio of CRS	44.51%	43.00%
Sichuan	Ratio of AMS	29.22%	73.86%
	Ratio of CRS	49.88%	83.20%

(3) **Micro-level fact: Characteristics of changes in farmland scale**

Table 4 shows the results of descriptive statistics of the large-scale plot. Generally, the scale of land parcels in Jiangsu experienced the fastest process. From 2013 to 2018, the average maximum land area in Jiangsu increased from 40.93 acres to 81.86 acres, 7.5 times and 8.4 times that of Sichuan. Not only is the absolute area of the largest plots in Sichuan small, but the process of plot scaling could also be faster. However, although the continuous process is slow in Jilin, the overall plot scale is still more significant. In 2013 and 2018, the maximum plot area reached over 30 acres, and the total area of plots with an area exceeding 15 acres reached over 1000 acres in both 2013 and 2018.

**Table 4:** Scale of farmland in the sample area.

Province	Maximum plot scale		Total area of farmland over 30 mu		Total area of farmland over 15 mu	
	2013	2018	2013	2018	2013	2018
Jiangsu	40.93	81.86	272.79	525.60	403.01	760.76
Sichuan	5.48	9.78	17.87	24.39	20.21	28.01
Jilin	34.73	35.06	266.48	269.66	1005.26	1039.05

(4) **Statistical description of other variables**

The statistical results show a highly correlated trend between the proportion of CRS and the process of land parcel scaling. In areas with larger land areas and faster scaling processes, CRS are shrinking, while in areas with smaller land areas and slower scaling processes, CRS still need to be developed. Of course, a more accurate causal relationship between the two still requires scientific and accurate econometric methods to test. Tables 5 and 6 provide descriptive statistical results for all other control variables at service providers and the village.

**Table 5:** Descriptive statistics of plot scaling and other control variables at the service providers.

Variables	sample size	Indicator Explanation/Unit	Mean	SD	Min	Max
The total area of plots of over 30 mu	564	mu	0.18	0.47	0	3
The total area of plots of over 15 mu	564	mu	0.23	0.51	0	3
Total area of plots of over 5 mu	564	mu	0.33	0.66	0	3.75
farm area	564	mu	156.3	249.4	0	2100
Plots		piece	18.04	21.40	0	100
The number of people in a family	564	population	4.49	1.65	1	11
Number of children aged over 16	564	population	0.83	0.85	0	4
The proportion of joint outsourcing service area	564	%	6.09	21.88	0	100
Whether it is inter-regional service	564	1 = Yes; 2 = No	0.25	0.43	0	1
Age of household head	188	years old	48.64	9.01	26	74
Gender of household head	188	1 = Male; 0 = Female	0.97	0.18	0	1
Household head's years of education	188	year	7.70	2.70	0	15
Health of household head	188	1 = Good; 0 = Poor	0.95	0.22	0	1
Party membership	188	1 = Yes; 0 = No	0.18	0.39	0	1
Cadre status	188	1 = Yes; 0 = No	0.16	0.37	0	1
Farming experience	188	year	22.92	12.11	0	53

Note: 1. Data source: Compiled based on research data from Jiangsu, Sichuan, and Jilin provinces.

**Table 6:** Descriptive statistical results of control variables at the village.

Variables	Indicator Explanation/Unit	Mean	SD	Min	Max
Total number of households	households	601.10	398.17	158	3100
Ageing rate	The proportion of population aged 60 and above to the total population of the village (%)	23.04	8.08	2	56
Feminization rate	The proportion of the female population to the total population of the village (%)	47.37	4.97	27	67
Share of nonfarm income	The proportion of non-agricultural income to total village income (%)	61.53	22.37	5	100
Total output value of village economy	billion yuan	19.01	105.52	0	965.8
The average wage of agricultural hired workers	yuan/day	105.93	29.54	0	250
Percentage of sloping land area of 15° and above	%	22.82	23.47	0	100
Number of plots per household	plots	5.92	3.53	1	25
The new rural endowment insurance coverage rate	%	82.33	20.82	2	100
Percentage of the number of farmers with the largest surname	%	20.73	17.18	0.5	95

Note: 1. Data source: Compiled based on research data from Jiangsu, Sichuan, and Jilin provinces. 2. The sample size is 584.

## 5. Results and discussion

### 5.1. Foundation model: The Impact of Plot Size on the Substitution of Local Services for CRS

Table 7 presents the results of the baseline model. The results show that plot scaling significantly negatively affects CRS. Specifically, as plot size increases, the proportion of farmland area serviced by CRS decreases. A 10-mu increase in the largest plot scale is associated with a 0.7% decrease in the CRS area proportion. A 10-mu increase in the total area of plots larger than 30 mu leads to a 0.1% decrease. A 10-mu increase in the total area of plots larger than 15 mu leads to a nearly 0.07% decrease.

**Table 7:** The results of the plot scale on the substitution of CRS.

Variables	The proportion of CRS area (FE)		
	(1)	(2)	(3)
Maximum plot scale	−0.070***		
	(−2.75)		
Total area of plots of 30 mu and above		−0.010**	
		(−2.29)	
Total area of plots of 15 mu and above			−0.007**
			(−2.43)
Total number of households	−0.013	−0.013	−0.013
	(−1.11)	(−1.13)	(−1.09)
Aging rate	0.043	0.034	0.023
	(0.33)	(0.26)	(0.17)
Feminization rate	−0.061	−0.055	−0.051
	(−0.29)	(−0.26)	(−0.24)
Net income per capita	0.543	0.446	0.485
	(0.30)	(0.25)	(0.27)
Share of nonfarm income	0.066	0.059	0.050
	(1.14)	(1.02)	(0.88)
Total output value of village economy	0.005	0.002	0.003
	(0.57)	(0.18)	(0.35)
Average wage of agricultural hired workers	0.06	0.06	0.06
	(1.48)	(1.50)	(1.53)
Number of plots per household	1.143***	1.167***	1.168***
	(2.67)	(2.72)	(2.72)
Percentage of sloping land area of 15° and above	−0.070	−0.066	−0.063
	(−1.29)	(−1.21)	(−1.15)
The new rural endowment insurance coverage rate	−0.061	−0.055	−0.054
	(−1.23)	(−1.10)	(−1.10)
Percentage of the number of farmers with the largest surname	0.086	0.096	0.094
	(0.94)	(1.04)	(1.03)
Year dummy variable	Control	Control	Control
Constant	13.847	13.345	14.838
	(0.96)	(0.92)	(1.03)
Number of samples	584	584	584
F-statistic value	5.41***	5.19***	5.26***
R-squared	0.201	0.195	0.197

Note: t-statistic values are in parentheses. ***, **, * denote 1%, 5%, 10% statistical significance levels, respectively.

### 5.2. Mechanisms: Cost and efficiency differences under scaling

To understand the mechanisms behind this substitution, we examine the differential impact of farm scale on the costs and efficiency of CRS versus local services.

We test the impact of farm scale on the cost and efficiency of CRS and local services. The results are shown in Tables 8 and Table 9, where columns (1), (2), and (3) denote the regression estimation results of “total area of 30 mu contiguous operation plots,” “total area of 15 mu contiguous operation plots,” and “total area of 5 mu contiguous operation plots” respectively.

**Table 8:** Plot scaling on cost of CRS and local services.

Variables	Cost per acre (FE)		
	(1)	(2)	(3)
Plots of land concentrated contiguous (10,000 mu)	−102.37***	−95.40***	−62.62***
	(−4.01)	(−4.05)	(−3.46)
Whether it is inter-regional service	−19.25	−24.07	−33.14**
	(−0.94)	(−1.18)	(−1.51)
Interaction term	67.02*	74.76**	38.17
	(1.78)	(2.33)	(1.53)
Control variables	Control	Control	Control
Constant	147.68***	148.91***	152.91***
	(3.24)	(3.27)	(3.31)
Observations	564	564	564
R^2^	0.004	0.003	0.002
F-statistic value	2.88***	2.89***	2.47***

Note: t-statistics are in parentheses, ***, **, and *, denoting 1%, 5%, and 10% statistical significance levels, respectively.

**Table 9:** Plot scaling on the efficiency of CRS and local services.

Variables	Efficiency (OLS)		
	(1)	(2)	(3)
Plots of land concentrated contiguous (10,000 mu)	0.157***	0.136**	0.110*
	(2.87)	(2.32)	(1.89)
Whether it is inter-regional service	0.012	0.014	0.016
	(0.81)	(0.93)	(1.02)
Interaction term	−0.141**	−0.123**	−0.103*
	(−2.45)	(−2.01)	(−1.74)
Control variables	Control	Control	Control
Constant	−0.005	−0.007	−0.004
	(−0.07)	(−0.11)	(−0.06)
Observations	188	188	188
R^2^	0.31	0.28	0.25

Note: t-statistics are in parentheses, ***, **, and *, denoting 1%, 5%, and 10% statistical significance levels, respectively.

#### 5.2.1. Cost differences.

The results in Table 9, based on fixed-effects models (supported by Hausman tests), show that plot scaling helps reduce the per-mu mechanization cost. However, this cost-reduction effect is significantly weaker for CRS compared to local services.

For instance, for every 1,000-mu increase in the total area of contiguous plots over 30 mu, the per-mu cost for local services decreases by approximately 10 yuan, whereas for CRS it decreases by only about 4 yuan. This pattern holds across other scaling measures (contiguous plots over 15 mu and 5 mu), indicating that local services realize greater economies of scale from land consolidation.

#### 5.2.2. Efficiency differences.

As shown in Table 9, plot scaling significantly improves the operational efficiency of farm mechanization. Crucially, the interaction term between plot scaling and the use of CRS is negative and significant. This indicates that while scaling benefits both service types, the efficiency gains are substantially larger for local services than for CRS. The efficiency advantage of local services grows as the scale of contiguous operational area increases.

### 5.3. Robustness test

#### 5.3.1. Subsample analysis by crop type.

We re-estimated the baseline model separately for rice and corn samples (Tables 10 and 11). The negative effect of plot scaling on CRS use is more pronounced for rice. For rice, the proportion of CRS will decrease by 1% for every 10 mu increase in the maximum plot scale and by about 0.2% for every 10 mu increase in the total area of plots over 30 mu. For each 10 mu increase in the total area of plots over 15 acres, the proportion of CRS will decrease by about 0.1%.

**Table 10:** The results of the plot scale on the substitution of CRS (rice).

Variables	The proportion of CRS area (FE)		
	(1)	(2)	(3)
Maximum plot	−0.102***		
	(−2.83)		
Total area of plots over 30 mu		−0.015***	
		(−2.67)	
Total area of plots over 15 mu			−0.012***
			(−2.78)
Control variables	Control	Control	Control
Number of samples	291	291	291
F-statistic value	5.57***	5.47***	5.54***
R-squared	0.372	0.368	0.371

Note: t-statistic values are in parentheses. ***, **, * denote 1%, 5%, 10% statistical significance levels, respectively.

**Table 11:** The result of the plot scale on the substitution of CRS (corn).

Variables	Proportion of inter-regional outsourcing services area (FE)		
	(1)	(2)	(3)
Maximum plot scale	−0.015		
	(−0.91)		
Total area of plots of over 30 mu		−0.005	
		(−0.47)	
Total area of plots of over 15 mu			0.001
			(0.46)
Control variables	Control	Control	Control
Number of samples	293	293	293
F-statistic value	2.56***	2.5***	2.5***
R-squared	0.213	0.209	0.209

Note: t-statistic values are in parentheses,***, **, and *, denoting 1%, 5%, and 10% statistical significance levels, respectively.

The relationship is not statistically significant for corn, likely due to its more complex agronomy and relatively slower mechanization progress, which may mute the scale effect.

#### 5.3.2. Addressing endogeneity.

To address potential endogeneity, we employed an instrumental variable (IV) approach. We used the number of grain procurement points five years prior as an instrument for the current level of scale farming, arguing it influenced historical support for scaling but is unrelated to current CRS choices except through scaling.

The instrumental variable (IV) regression results are presented in Table 12. The first-stage results confirm a statistically significant positive relationship between the historical level of support services for scale farming—instrumented by the number of grain procurement points five years prior—and the current scale farming level (Coefficient = 7.14, t = 3.80). The F-statistic of 14.41 (p < 0.01) from the weak instrument test is well above the conventional threshold of 10, indicating that the chosen instrument is strong and that weak instrument concerns are not present.

**Table 12:** The Results of Instrumental Variable Estimation.

Variables	First-Stage Results (Scale Farming)		Second-Stage Results (CRS Area Proportion)	
	Coefficient	t-stat	Coefficient	z-stat
the number of grain procurement points in the village five years prior	7.14***	3.80		
Maximum plot scale			−0.35**	−2.35
Total number of households	0.03***	3.65	0.02***	3.31
Aging rate	0.66**	2.19	0.63***	3.33
Feminization rate	0.23	0.49	−0.30	−1.12
Net income per capita	5.27	1.26	9.95***	4.01
Share of nonfarm income	−0.24**	−2.08	0.10	1.36
Total output value of village economy	0.01	0.27	0.01	0.44
Average wage of agricultural hired workers	0.06	0.72	0.10**	2.23
Number of plots per household	−1.12	−1.48	1.38***	2.98
Percentage of sloping land area of 15° and above	−0.28***	−2.58	−0.20***	−2.67
The new rural endowment insurance coverage rate	−0.01	−0.07	0.13*	1.93
Percentage of the number of farmers with the largest surname	0.08	0.55	0.11	1.26
Year dummy variable	Control		Control	
Constant	−5.50	−0.20	−28.47	−1.85
Number of samples	584		584	
F-statistic	14.41***			

Note: ***, **, and *, denoting 1%, 5%, and 10% statistical significance levels, respectively.

The second-stage regression results demonstrate that, after accounting for endogeneity and controlling for other covariates, the scale farming level (measured by maximum plot area) continues to exert a statistically significant negative effect on the proportion of CRS area (Coefficient = −0.35, z = −2.35). This finding is consistent with the baseline regression estimates, reinforcing the robustness of the core conclusion.

### 5.4. Discussion

The analysis demonstrates that farm scaling enhances operational efficiency and reduces costs in farm mechanization. However, the marginal effect on cost and efficiency of local services and CRS are different, and the scaling of farms has a more significant effect on local services than on CRS. As the comparative advantage of local services becomes increasingly evident, they exert competitive pressure on CRS, leading to a gradual displacement of CRS by self-owned machinery in recent years—a trend particularly observable in China.

This dynamic suggests that local services will likely replace CRS in regions with high levels of farmland consolidation, whereas CRS may persist in areas with smaller, fragmented plots, where it helps mitigate labor shortages and high production costs. Nevertheless, the dominant trajectory of farm mechanization in China points toward the growing prevalence of local services, a conclusion aligned with the findings of Liu et al. [23] and Li & Luo [25].

This trend mirrors the mechanization patterns observed in developed countries such as the United States, where large-scale, consolidated farmland promotes the dominance of self-owned machinery and local service provision. Conversely, the insights from this study also hold relevance for low- and middle-income countries where small and fragmented landholdings remain prevalent.

Furthermore, existing studies indicate that local agricultural machinery services are often characterized by inferior equipment quality, which may lead to a low-level equilibrium in this market. Notably, the provision of localized agricultural machinery services could also trigger a “non-grain” cultivation tendency in village farmlands. However, there remains a paucity of systematic empirical research regarding the multidimensional impacts brought about by the localization of agricultural machinery services.

## 6. Conclusions

### 6.1. Summary

This study addresses the changes in the forms of farm mechanization for grain production in China. It constructs an analytical framework concerning the theory of specialized division of labor and the theory of scale economy. At the theoretical level, we systematically analyze the influence and mechanism of farmland scaling on farm mechanization forms and conduct empirical tests to systematically and deeply explain the causes and micromechanisms of the development and changes of farm mechanization forms of grain production in China.

The conclusion is that Scaling of land parcels promotes substituting local services for CRS by changing their economies of scale advantages. Although the scale of land parcels can help improve the operating conditions for local services and CRS, their marginal effects on increasing efficiency and reducing cost differ. The marginal effect of local services is greater than that of CRS. This means that the increase in local services such as self-purchasing agricultural machinery will not reduce the efficiency of grain production.

This conclusion is in line with the current situation in China. China's CRS are already mainly operated on larger plots of land, and the scale of the plots within a specific range has a limited impact on them. For local services, the scale of the plot may mean that the area was previously unable to use machinery and could shift towards using small and medium-sized machinery. Therefore, adjusting machine inputs based on the number and size of land parcels to achieve optimal allocation of agricultural production factors has higher flexibility. At the same time, due to the advantages of the timely agricultural season, rural social and human relations, etc., it can also seize the market on contiguous plots of land that inter-regional service providers originally completed. In summary, the scale of land parcels has a greater marginal efficiency-enhancing effect on local services.

With the advancement of large-scale agriculture in China, the fragmentation of land parcels in various regions has changed. And this will promote the widespread rise of local services while CRS will further decrease.

### 6.2. Policy implications

We can draw the following policy recommendations based on the above conclusions and combined with the current social reality of China's agricultural development stage.

(1) Adapt Policies to the Phased Characteristics of AMS Market Transformation

At different stages of the agricultural machinery service market development, policies should be dynamically adjusted to meet evolving demands. For instance, during the rapid growth period of cross-regional services, the policy focus should shift toward promoting contiguous farming and establishing efficient trading platforms for cross-regional machinery services to optimize resource allocation. When cross-regional services face transaction risks and uncertainties, policies should encourage large-scale operators to expand land holdings, support the procurement of machinery and localized services, and strengthen industry regulation and service standardization to improve overall service quality.

(2) Balance Cross-Regional and Local Services to Build a Dual-Driven System of “Land Intensification + Service Socialization”

While localized agricultural machinery services are inevitable and rational, the specialization and economies of scale offered by cross-regional services remain irreplaceable. For example, Jiangsu has established a highly organized and market-mature cross-regional service system. Continued support for its cross-regional operations is of strategic significance in enhancing production efficiency in key grain-producing regions nationwide.

Therefore, a dual-track policy approach is essential: On one hand, cross-regional services should be optimized through policy support, technological upgrades, and information platform development to reduce operational uncertainties and consolidate their professional advantages, thereby improving efficiency in grain-producing regions. On the other hand, local service capabilities should be enhanced. Although local services excel in disaster response and operational stability, their specialization and technical efficiency still require improvement. For areas where contiguous farming is challenging or not covered by cross-regional service networks, organizational models should be refined, and factor markets improved to leverage the flexibility of local services while addressing efficiency gaps.

(3) Innovate Land Scale Management Models to Strengthen the Foundation for AMS Transformation

Although China's agricultural land scale management continues to advance, the growth rate has slowed. Thus, innovating land scale management models is crucial to driving the transformation of agricultural machinery services. In regions with abundant land resources, efforts should focus on land consolidation and connectivity. In areas with limited land resources, agricultural infrastructure construction and land improvement—such as building field roads and drainage channels—should be prioritized to ensure machinery accessibility and create favorable conditions for mechanized operations.

### 6.3. Limitations and future work

The change in the external environment is the main reason for transforming the farm mechanization mode of grain production. This article only discusses theoretical and empirical issues from the perspective of large-scale agricultural land management. In fact, in recent years, the agricultural production environment in China has undergone significant changes in many aspects. This is reflected in the agricultural production factors of the land and the constantly changing production factors and resource endowments such as population, capital, and information. Moreover, the relative cost prices of each production factor are constantly changing. All of these may significantly impact the changes in the grain production farm mechanization forms. However, this study did not analyze them individually due to limitations in ability and time, and further exploration is needed.

## Supporting information

S1 Fig This figure illustrates how changes in farm scale are associated with variations in farm modernization forms. Cost curves and average cost curves are shown across three modernization pathways: self-purchasing machinery (self), specialized local services (special), and cross-regional services (CRS). The horizontal axis represents increasing farm scale from L1 to Lm. The vertical axis captures cost values, with distinct curves indicating different cost structures for each pathway. The figure demonstrates that as farm scale increases from a small size (L1) to a larger size (L2), the relative cost advantage shifts. For small farms (e.g., at size L1), the high fixed costs of owning machinery make CRS the more economical choice. As the farm scale grows (e.g., to L2), the fixed costs of self-owned machinery are spread over a larger area, improving its cost-effectiveness. Eventually, for sufficiently large farm sizes, owning machinery or using local services becomes more economical than CRS. Furthermore, farmers who own machinery can also offer services to neighboring plots, achieving efficient scale at a lower total operational area than required for CRS to be viable.
(TIF)

S1 Data Data.
(XLSX)
